# A Single-arm, Multicenter, Phase 2 Study of Lenvatinib Plus Everolimus in Patients with Advanced Non-Clear Cell Renal Cell Carcinoma

**DOI:** 10.1016/j.eururo.2021.03.015

**Published:** 2021-04-16

**Authors:** Thomas E. Hutson, M. Dror Michaelson, Timothy M. Kuzel, Neeraj Agarwal, Ana M. Molina, James J. Hsieh, Ulka N. Vaishampayan, Ran Xie, Urmi Bapat, Weifei Ye, Rohit K. Jain, Mayer N. Fishman

**Affiliations:** a Texas Oncology-Baylor Charles A. Sammons Cancer Center, Dallas, TX, USA; b Massachusetts General Hospital Cancer Center, Boston, MA, USA; c Rush University Medical Center, Chicago, IL, USA; d Huntsman Cancer Center, (NCI-CCC), University of Utah, Salt Lake City, UT, USA; e Weill Cornell Medicine, New York, NY, USA; f Washington University School of Medicine, St. Louis, MO, USA; g Barbara Ann Karmanos Cancer Institute, Detroit, MI, USA; h Eisai Inc., Woodcliff Lake, NJ, USA; i Green Key Resources, LLC, New York, NY USA; j H. Lee Moffitt Cancer Center, Tampa, FL, USA; k Cancer Center of South Florida, Tampa, FL, USA

**Keywords:** Non-clear cell renal cell carcinoma, Chromophobe, Papillary, Everolimus, First-line treatment, Lenvatinib

## Abstract

**Background:**

Non-clear cell renal cell carcinoma (nccRCC) accounts for ≤20% of RCC cases. Lenvatinib (a multitargeted tyrosine kinase inhibitor) in combination with everolimus (an mTOR inhibitor) is approved for the treatment of advanced RCC after one prior antiangiogenic therapy.

**Objective:**

To determine the safety and efficacy of lenvatinib plus everolimus as a first-line treatment for patients with advanced nccRCC.

**Design, setting, and participants:**

This open-label, single-arm, multicenter, phase 2 study enrolled patients with unresectable advanced or metastatic nccRCC and no prior anticancer therapy for advanced disease.

**Intervention:**

Lenvatinib (18 mg) plus everolimus (5 mg) orally once daily.

**Outcome measurements and statistical analysis:**

The primary endpoint was the objective response rate (ORR) as assessed by investigators according to Response Evaluation Criteria in Solid Tumors version 1.1. Secondary endpoints included progression-free survival (PFS), overall survival (OS), and safety assessments. The 95% confidence intervals (CIs) for ORRs were calculated using the two-sided Clopper-Pearson method. Median PFS and median OS were estimated using the Kaplan-Meier product-limit method and their 95% CIs were estimated via a generalized Brookmeyer and Crowley method.

**Results and limitations:**

The study (start date: February 20, 2017) enrolled 31 patients with nccRCC (papillary, *n* = 20; chromophobe, *n* = 9; unclassified, *n* = 2). At the data cutoff date (July 17, 2019), the best overall response was a partial response (eight patients: papillary, *n* = 3; chromophobe, *n* = 4; unclassified, *n* = 1) for an overall ORR of 26% (95% CI 12–45). Median PFS was 9.2 mo (95% CI 5.5–not estimable), and median OS was 15.6 mo (95% CI 9.2–not estimable). The most common treatment-emergent adverse events were fatigue (71%), diarrhea (58%), decreased appetite (55%), nausea (55%), and vomiting (52%). Limitations include the small sample size and single-arm design.

**Conclusions:**

Lenvatinib plus everolimus showed promising anticancer activity in patients with advanced nccRCC with an ORR of 26% and is worthy of further study. The safety profile was consistent with the established profile of the study-drug combination.

**Patient summary:**

We examined the combination of lenvatinib plus everolimus as the first therapy for 31 patients who had advanced nccRCC. We found that this treatment seemed effective, because most patients had a decrease in tumor size and manageable treatment-related side effects.

**Clinical registration:**

This trial is registered at ClinicalTrials.Gov as NCT02915783.

## Graphical Abstract

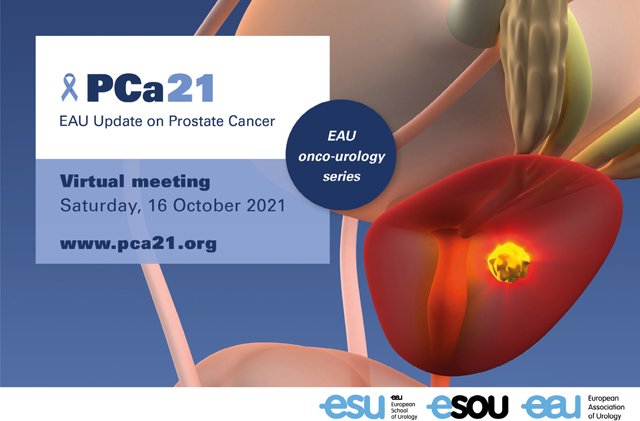

## 1. Introduction

Renal cell carcinoma (RCC) is generally grouped into two principal subtypes: clear cell RCC (ccRCC), which accounts for more than 80% of RCC cases, and non-clear cell RCC (nccRCC), an umbrella term that encompasses the remaining histological subtypes [1]. The histological subtypes that fall under the nccRCC designation include papillary RCC, chromophobe RCC, unclassified RCC, collecting duct carcinoma, and renal medullary carcinoma, among others [1,2].

Historically, the majority of RCC clinical trials have focused on ccRCC or mixed RCC populations. However, over the past five years, studies have begun to specifically enroll patients with nccRCC, and treatments with single-agent VEGF and mTOR inhibitors have been assessed in this patient population. Disappointingly, compared to the response rates observed for patients with ccRCC, these studies reported low response rates, with overall objective response rates (ORRs) ranging from 3% to 18% [3,4].

The mTOR pathway has been implicated in the pathogenesis of RCC, with mutations in this pathway occurring at a frequency of approximately 5% in both the chromophobe and papillary subtypes of nccRCC [5]. A phase 2 clinical study of patients with nccRCC and no prior systemic therapy compared the effectiveness of sunitinib (a VEGF-targeted tyrosine kinase inhibitor [TKI]) and the mTOR inhibitor, everolimus [3]. Interestingly, among patients with chromophobe nccRCC in the study, 33% (*n* = 2/6) had an objective response to everolimus treatment, compared to 10% (*n* = 1/10) with sunitinib. By contrast, patients with papillary nccRCC fared better with sunitinib treatment than with everolimus, with ORRs of 24% (*n* = 8/33) and 5% (*n* = 2/37), respectively [3]. In a phase 2 clinical study of 34 patients with different histologic subtypes of nccRCC treated with a combination of bevacizumab and everolimus, the ORR was 29% [6]. Taken together, these data suggest that both VEGF- and mTOR-directed agents may be effective therapies for multiple nccRCC subtypes and further study is warranted.

Lenvatinib, in combination with everolimus, is approved for the treatment of patients with advanced RCC following one prior antiangiogenic therapy [7]. Lenvatinib is a multitargeted TKI of VEGF receptors 1–3, FGF receptors 1–4, platelet-derived growth factor receptor α, RET, and KIT [8–11]. The combination of lenvatinib plus everolimus has shown enhanced antitumor activity in patients with RCC [12,13] in both clinical and real-world settings [13,14]. Preclinical experiments have demonstrated that lenvatinib plus everolimus yields enhanced inhibition of both VEGF- and FGF-driven angiogenesis that is greater than for either agent alone [15]. Thus, it is hypothesized that dual inhibition of both the VEGF- and FGF-driven pathways, and downstream mTOR pathways, using the combination of lenvatinib plus everolimus, may contribute to the enhanced inhibition of both angiogenic and proliferation pathways in RCC [12,15].

While both VEGF-targeted TKIs and everolimus have shown some promise as monotherapies for nccRCC, it is not known whether targeting both pathways simultaneously will confer a greater benefit for these patients. This phase 2 single-arm, multicenter study evaluated the safety and efficacy of lenvatinib (18 mg once daily) plus everolimus (5 mg once daily) in patients with unresectable advanced or metastatic nccRCC who had not received any prior anticancer therapy for advanced disease.

## 2. Patients and methods

### 2.1. Study design and patients

This phase 2 single-arm, multicenter study enrolled patients with histologically confirmed nccRCC (per study investigator), measurable disease per Response Evaluation Criteria In Solid Tumors version 1.1 (RECIST v1.1), and no prior anticancer therapy for advanced disease. Eligible patients must have had one of the following types of nccRCC: papillary, chromophobe, collecting duct carcinoma, renal medullary carcinoma, or unclassified RCC. In addition, eligible patients were required to have an Eastern Cooperative Oncology Group performance status (ECOG PS) score of 0 or 1, adequate liver, renal, and bone marrow function, and adequately controlled blood pressure (≤140/90 mm Hg). Patients were excluded according to the following key criteria: predominantly ccRCC, prior exposure to lenvatinib or an mTOR inhibitor, or major surgery ≤3 wk from the starting dose. Patients were also excluded if they had uncontrolled diabetes, proteinuria (urine protein, ≥1 g/24 h), interstitial lung disease or active noninfectious pneumonitis, or any condition that would affect the absorption of the study-drug combination.

Enrolled patients received lenvatinib (18 mg orally once daily) plus everolimus (5 mg orally once daily) in continuous 28-d cycles. Patients continued to receive one or both study drugs for as long as evidence of clinical benefit was present, or until intercurrent illness, unacceptable toxicity, disease progression, or withdrawal of patient consent.

The study was conducted in full accordance with the International Conference on Harmonization Good Clinical Practice guidelines and federal regulations. The protocol was approved by the institutional review board or independent ethics committee in each center. Written informed consent was obtained from all study participants before study enrollment. This trial is registered at ClinicalTrials.Gov as NCT02915783.

### 2.2. Study endpoints and assessments

The primary endpoint was the ORR as assessed by investigators using RECIST v1.1. Confirmation of complete response and partial response was required ≥4 wk after a response was first documented. Secondary endpoints included progression-free survival (PFS) and overall survival (OS). PFS and ORR by independent imaging review (IIR) were also assessed as exploratory endpoints. Additional exploratory endpoints included the clinical benefit rate (CBR; defined as the proportion of patients with a best overall response of complete response, partial response, or durable [≥23 wk] stable disease) and the disease control rate (DCR; defined as the proportion of patients with a best overall response of complete response, partial response, or stable disease). Tumor assessments were performed according to RECIST v1.1, with imaging studies carried out every 8 wk (±1 wk) after the first dose of study treatment.

Safety was assessed by monitoring and recording all adverse events, including all grades according to Common Terminology Criteria for Adverse Events (CTCAE) version 4.03.

### 2.3. Statistical analysis

At the time of study protocol development, the response rates for patients with nccRCC treated with the study drugs (lenvatinib and everolimus, either as monotherapies or in combination) were not available. The sample size for the study was calculated using Simon’s two-stage design for the primary endpoint of ORR assuming an ORR of 25% from this study versus a historical control of 8% for patients with advanced RCC [16]. A total of approximately 31 patients, including 16 in stage 1, were planned to be enrolled in the study. If there were one or no responders in stage 1, the enrollment would be stopped; if there were two or more responders, the study would proceed to stage 2. At interim analysis, based on an assumption of ORR = 8% for the null hypothesis and ORR ≥ 25% for the alternative hypothesis, the probability of early futility stopping was 0.6299 and 0.0635, respectively. This design would yield a one-sided type I error of 0.0319 and power of 0.8053 in stages 1 and 2 combined. In the final analysis, if six of 31 patients were considered responders, then the study ORR would be considered statistically significant compared with historical controls.

The 95% confidence intervals (CIs) for response rates were calculated using the two-sided Clopper-Pearson method. Median PFS and median OS were estimated using the Kaplan-Meier product-limit method and their 95% CIs were estimated with a generalized Brookmeyer and Crowley method.

## 3. Results

This phase 2 study enrolled 31 patients with nccRCC, all of whom received the study treatment. Most patients had papillary type (*n* = 20/31; 65%), followed by chromophobe, (*n* = 9/31; 29%), and unclassified (*n* = 2/31; 6%) nccRCC. Among the enrolled patients, the median age was 64 yr, and approximately two-thirds were men (65%). Most patients had an ECOG PS of 0 (*n* = 23/31; 74%), and fewer than half had undergone a prior nephrectomy (*n* = 11/31; 35%). Lymph nodes were the most common site of metastasis (*n* = 22/31; 71%; Table 1).

By the data cutoff date (July 17, 2019), 25 patients (81%) had discontinued the study treatment and six (19%) remained on treatment. The following reasons led to study-drug discontinuation: radiological or clinical disease progression (*n* = 15/31; 48%), adverse event (*n* = 6/31; 19%), or patient choice (*n* = 4/31; 13%). Among the 25 patients who discontinued treatment, eight remained on study follow-up for OS.

**Fig. 1 –:**
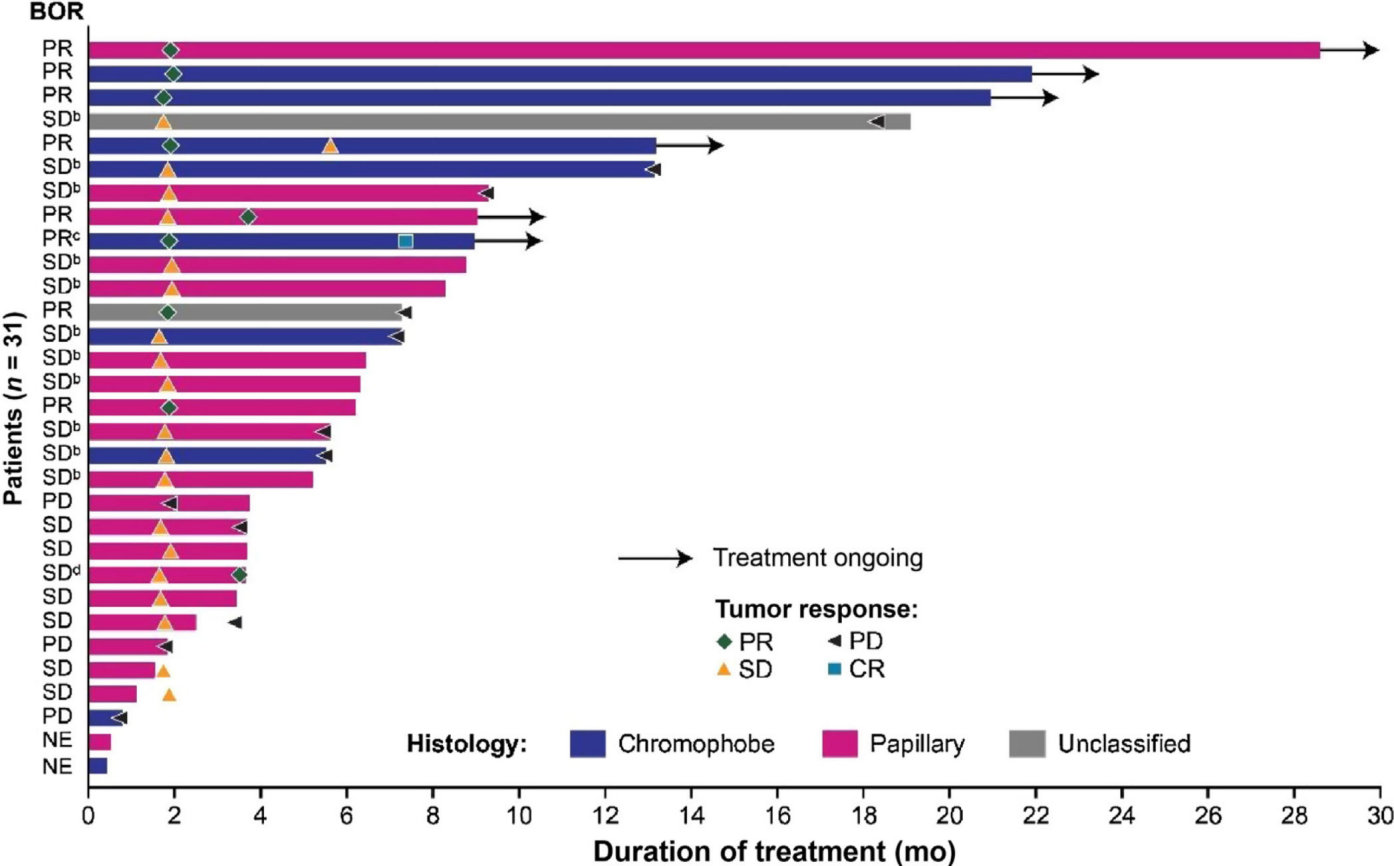
Duration of treatment and tumor response by investigator assessment according to Response Evaluation Criteria in Solid Tumors version1.1.^a^ BOR = best overall response; CR = complete response; PD = progressive disease; PR = partial response; NE = not evaluable; SD = stable disease. ^a^ The figure indicates the outcome at a patient’s first tumor assessment and then any subsequent change in tumor response status. ^b^ These patients had durable stable disease. ^c^ This patient had a complete response, but this response was not confirmed by the time of data cut-off, so the BOR for this patient is a partial response. ^d^ This patient had stable disease at first assessment and a subsequent partial response; since the partial response was not confirmed because of treatment discontinuation (patient’s choice), the BOR for this patient is stable disease.

A best overall response of partial response was observed in eight patients and no patients had a confirmed complete response (Fig. 1 and Table 2). The overall ORR was 26% (95% CI 12–45), both when assessed by an investigator and by IIR. As assessed by an investigator, stable disease was observed in 18 patients, for a DCR (complete response, partial response, or stable disease) of 84%, and durable (≥23 wk) stable disease was observed in 11 patients, for a CBR (complete response, partial response, or durable stable disease) of 61%. By comparison, the DCR and CBR by IIR were 71% and 52%, respectively (Table 2). The median duration of response was not estimable (NE); however, per IIR, the majority (88%) of responders (ie, those with complete or partial responses) had maintained their response for 5 mo.

Among 20 patients with papillary RCC, three had partial responses, for an ORR of 15% (*n* = 3/20), and an additional 14 patients had stable disease, for a DCR of 85% (*n* = 17/20; as assessed by investigator). A best overall response of partial response was observed in four patients with chromophobe nccRCC, resulting in an ORR of 44% (*n* = 4/9), and an additional three patients had stable disease, for a DCR of 78% (*n* = 7/9; as assessed by an investigator). Of the two patients in this study with unclassified nccRCC, one had a partial response and one had stable disease.

**Fig. 2 –:**
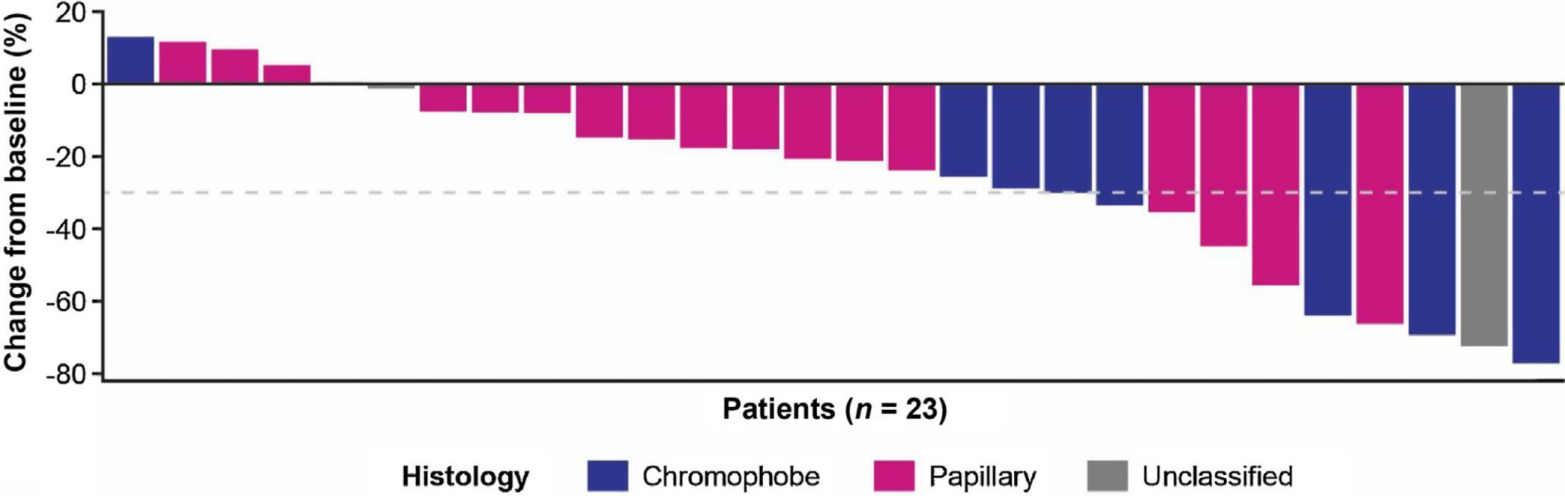
Percentage change in total sum of target lesion diameters from baseline to postbaseline nadir by investigator assessment according to Response Evaluation Criteria in Solid Tumors version 1.1. The analysis included patients with both baseline and at least one postbaseline target lesion assessment.

**Fig. 3 –:**
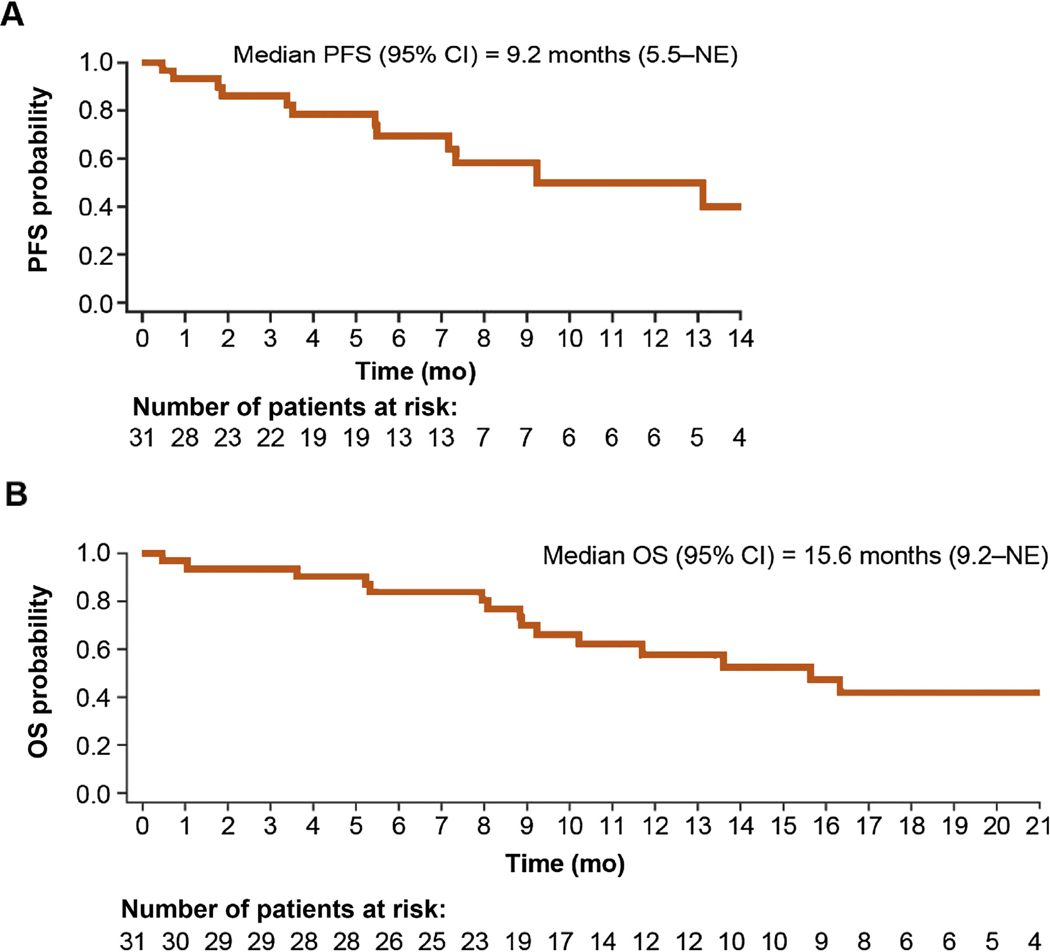
Kaplan-Meier estimates of (A) PFS and (B) OS by investigator assessment according to Response Evaluation Criteria in Solid Tumors version 1.1. The survival estimates were truncated when fewer than five patients at risk remained. CI = confidence interval; NE = not estimable; OS = overall survival; PFS = progression-free survival.

Most patients had a decrease in tumor size both by investigator assessment (Fig. 2) and by IIR (Supplementary Fig. 1). The median PFS was 9.2 mo (95% CI 5.5–NE) by investigator assessment (Fig. 3A) and 5.6 mo (95% CI 3.5–NE) by IIR. Median OS was 15.6 mo (95% CI 9.2–NE; Fig. 3B). The median PFS by investigator assessment and OS outcomes by histological subtypes are shown in Table 2.

All 31 patients experienced at least one treatment-emergent adverse event (TEAE). The five most common TEAEs (any grade) were fatigue (71%), diarrhea (58%), decreased appetite (55%), nausea (55%), and vomiting (52%; Table 3). TEAEs of grade ≥3 severity occurred in 68% of patients (*n* = 21/31). TEAEs led to study-drug discontinuation or withdrawal by 32% of patients (*n* = 10/31; one each for cardiac arrest, cardiac failure, arthralgia, back pain, cancer pain, hepatic encephalopathy, and tremor; and three for malignant neoplasm progression). TEAEs led to a dose reduction (lenvatinib only) in 45% (*n* = 14/31), and study-drug interruption (lenvatinib and/or everolimus) in 68% (*n* = 21/31) of patients. Overall, patients had a median relative dose intensity of 87% (range 32–100%) for lenvatinib and 94% (range 64–100%) for everolimus.

Overall, treatment-related TEAEs (all grades) occurred in 94% of patients, and 48% (*n* = 15/31) of patients had at least one treatment-related TEAE of grade ≥3 severity. Although there were three fatal TEAEs (malignant neoplasm progression, *n* = 2; cardiac arrest, *n* = 1), these were assessed as not related to treatment by the investigators.

## 4. Discussion

This phase 2 study demonstrated that the combination of lenvatinib plus everolimus showed promising antitumor activity as a potential first-line therapy for patients with advanced nccRCC, with an overall ORR of 26% (both by investigator assessment and by IIR). The observed overall ORR of 26% in this study meets the prespecified threshold for statistical significance compared to historical controls of patients with advanced RCC (available at the time of study protocol development). Moreover, the ORR of 26% compares favorably to current response rates reported for patients with nccRCC who were treated with everolimus monotherapy (9%) [3]. While no ORRs have been reported to date for patients with nccRCC who were treated with single-agent lenvatinib, the observed ORR (26%) appears higher than that seen with other single-agent TKIs (ie, sunitinib; ORRs range from 9% to 18% [3,4]) and comparable to overall ORRs reported for patients with nccRCC in response to immunotherapy (ORRs range from 10% to 26% [17–21]). While no confirmed complete responses were observed in this study, partial responses were observed in patients across all histological subtypes enrolled (papillary, chromophobe, and unclassified nccRCC).

Historically, ORRs for patients with nccRCC have been low (3–18%) [3,4]. Thus, more recent studies have been aimed at improving outcomes in these patients. Retrospective analyses for both cabozantinib and nivolumab have shown some potential in patients with nccRCC, with overall ORRs of 22–27% recorded [17,22]. However, prospective studies for cabozantinib in nccRCC are still lacking. Moreover, a clinical trial among patients with nccRCC who were treated with nivolumab demonstrated a range of responses based on histological subtypes (ORRs ranging from 0% to 25%) [18], suggesting that response to nivolumab may be subtype-specific. Similarly, in a recent single-arm, phase 2 study of first-line pembrolizumab among 165 patients with nccRCC (KEYNOTE-427B) [19], the ORRs observed ranged between 10% and 31% for different histological subtypes.

Additional studies have been conducted in patients with specific papillary nccRCC subtypes. Srinivasan et al [23] reported on a phase 2 study of patients with type 2 papillary nccRCC who were treated with bevacizumab and erlotinib, and demonstrated an ORR of 51% (*n* = 42/83; 95% CI 40–61). The phase 3 SAVIOR trial, which assessed the efficacy of savolitinib (a MET-kinase inhibitor) versus sunitinib in patients with metastatic MET-driven papillary nccRCC, reported an ORR of 27% (*n* = 9/33; 95% CI 13–46) in the savolitinib treatment arm and an ORR of 7% (*n* = 2/27; 95% CI 1–24) in the sunitinib arm [24]. While these data appear promising for patients with these specific subtypes of nccRCC (eg, type 2 papillary or MET-driven papillary), additional therapies are still needed for patients with histological subtypes that were not investigated in these trials. Moreover, enrollment in clinical trials is still a recommended treatment option for patients with nccRCC [25]; consequently, further prospective studies are warranted for patients with nccRCC to identify better treatments across histological subtypes.

Among 20 patients with papillary histology treated with lenvatinib plus everolimus in the current study, three had partial responses for an ORR of 15% (*n* = 3/20; 95% CI 3–38). By comparison, an ORR of 24% was observed in papillary nccRCC treated with single-agent sunitinib [3]. Notably, in our study four patients with chromophobe nccRCC had a partial response for an ORR of 44% (*n* = 4/9; 95% CI 14–79), and one of the two patients with unclassified histology also had a partial response. By comparison, in the KEYNOTE 427B study [19], promising ORRs of 28% (95% CI 20–37) and 31% (95% CI 14–52) were observed for papillary and unclassified nccRCC subtypes, respectively· However, a lower ORR of 10% (95% CI 1–30) was observed for the chromophobe histological subtype. Taken together, the data in the current study suggest that the combination of lenvatinib plus everolimus may hold promise across histological subtypes, with some subtypes appearing to derive more benefit. The subtypes that respond best to PD-1–targeted immunotherapy versus the multitargeted lenvatinib plus everolimus combination appear divergent, which suggests that further studies may be warranted to distinguish between these agents and the nccRCC subtypes that would benefit the most from these treatments.

Particularly noteworthy in this study is the promising ORR of 44% (*n* = 4/9; 95% CI 14–79) and CBR of 78% (*n* = 7/9; 95% CI 40–97) for patients with chromophobe nccRCC. Although this study is limited by its small sample size (*n* = 31 overall and *n* = 9 for the chromophobe histology) and cross-study comparisons have inherent limitations, these results compare favorably to the ORRs ranging from 10% to 33% observed among patients with chromophobe nccRCC with single-agent sunitinib or everolimus treatment in the ASPEN study [3]. Moreover, the ORR of 44% in the current study is higher than the ORR range of 0–10% for patients with chromophobe nccRCC treated with immunotherapy [17–19].

The mTOR pathway has been implicated in the development of chromophobe nccRCC. Patients with Birt-Hogg-Dube syndrome develop a hereditary form of chromophobe nccRCC characterized by the development of tumors with highly active PI3K/mTOR pathways [26]. While further studies may be needed to fully delineate the mechanism of action that accounts for the apparent enhanced anticancer activity of lenvatinib plus everolimus in this histological subtype, our study results are consistent with the hypothesis that dual inhibition of both the VEGF- and FGF-driven pathways, as well as downstream mTOR pathways, by this combination [12,15], leads to enhanced antitumor activity in patients with nccRCC. In addition, as the mTOR pathway is implicated in the pathogenesis of chromophobe nccRCC, inhibition of mTOR via everolimus may account for the enhanced anticancer activity observed in this subtype in the current study.

With respect to the secondary endpoints in the current study, treating patients with nccRCC with lenvatinib plus everolimus resulted in an overall median OS of 15.6 mo, which was similar to that observed with first-line treatment with single-agent sunitinib (16.2 mo) or everolimus (14.9 mo) [4]. While the median OS was similar, the median PFS of 9.2 mo was encouraging and appeared prolonged compared with that observed with first-line sunitinib (6.1 mo) or everolimus (4.1 mo) treatment [4]. Moreover, it is noteworthy that the median PFS was 9.2 mo in the current study, even though 61% of patients had two or more sites of metastases and the majority of patients (87%) were in the intermediate or poor prognostic risk group.

Given the pathways involved in nccRCC and the mechanisms of action of lenvatinib and everolimus, further applied development of these combined agents and combinations of agents with other mechanisms, such as anti–PD-1/L1 plus anti–CTLA4 antibodies or anti–PD-1/L1 agents plus VEGF inhibitors remain of interest.

## 5. Conclusions

This open-label, phase 2 study of the combination of lenvatinib plus everolimus as a first-line treatment for patients with advanced or metastatic nccRCC achieved an ORR of 26% (95% CI 12–45) by both investigator assessment and IIR, with an ORR of 44% among patients with chromophobe histology. The combination demonstrated encouraging anticancer activity, with a median OS of 15.6 mo, and median PFS of 9.2 mo by investigator assessment and 5.6 mo by IIR. The tolerability profile observed in this study was similar to the established safety profiles of the study-drug combination in RCC [13], with no new safety signals. Cumulatively, these data suggest that the combination of lenvatinib plus everolimus has promising anticancer activity and is worthy of future study in patients with nccRCC.

## Supplementary Material

Supplementary Material

## Figures and Tables

**Table 1 –:** Baseline patient demographics and disease characteristics for the 31 study participants

Parameter	Result

Median age, yr (range)	64 (38–85)
Males, *n* (%)	20 (65)
Race, *n* (%)	
White	27 (87)
Black or African American	1 (3)
Other	3 (10)
ECOG performance status 0, *n* (%) ^a^	23 (74)
Histology, *n* (%)	
Papillary	20 (65)
Chromophobe	9 (29)
Unclassified	2 (6)
Sites of metastases at baseline, *n* (%)	
Adrenal	4 (13)
Bone	7 (23)
Liver	9 (29)
Lung	8 (26)
Lymph node	22 (71)
Other	11 (35)
Number of metastatic sites, *n* (%)	
0	0
1	12 (39)
2	10 (32)
≥3	9 (29)
Prior nephrectomy, *n* (%)	11 (35)
IMDC prognostic group at baseline, *n* (%)	
Favorable risk	4 (13)
Intermediate risk	20 (65)
Poor risk	7 (23)

ECOG = Eastern Cooperative Oncology Group; IMDC = International Metastatic Renal Cell Carcinoma Database Consortium.

a The remaining patients had ECOG performance status of 1, in accordance with the protocol.

**Table 2 –:** Summary of efficacy outcomes by histological subtype

Parameter	By investigator assessment				By IIR
	Papillary (*n* = 20)^a^	Chromophobe (*n* = 9)^a^	Unclassified (*n* = 2)^a^	Total (*n* = 31)	Total (*n* = 31)

Objective response rate, *n* (%) (95% CI) ^b^	3 (15) (3–38)	4 (44) (14–79)	1 (50) (1–99)	8 (26) (12–45)	8 (26) (12–45)
Best overall response, *n* (%)					
Complete response	0	0	0	0	0
Partial response	3 (15)	4 (44)	1 (50)	8 (26)	8 (26)
Stable disease	14 (70)	3 (33)	1 (50)	18 (58)	14 (45)
Durable stable disease ^c^	7 (35)	3 (33)	1 (50)	11 (35)	8 (26)
Progressive disease	2 (10)	1 (11)	0	3 (10)	6 (19)
Not evaluable/unknown	1 (5)	1 (11)	0	2 (6)	3 (10)
Clinical benefit rate, n (%) ^d^	10 (50)	7 (78)	2 (100)	19 (61)	16 (52)
(95% CI) ^b^	(27–73)	(40–97)	(16–100)	(42–78)	(33–70)
Disease control rate, n (%) ^e^	17 (85)	7 (78)	2 (100)	26 (84)	22 (71)
(95% CI) ^b^	(62–97)	(40–97)	(16–100)	(66–95)	(52–86)
Median PFS, mo (95% CI)^f,g^	9.2 (3.5–NE)	13.1 (0.5–NE)	12.8 (7.3–18.3)	9.2 (5.5–NE)	5.6 (3.5–NE)
Median OS, mo (95% CI) ^f,g^	11.7 (8.1–NE)	NE (0.5–NE)	NE (NE–NE)	15.6 (9.2–NE)	NA

CI = confidence interval; IIR = independent imaging review; NA = not applicable; NE = not estimable; OS = overall survival; PFS = progression-free survival.

a Percentages for the histological subtypes (papillary, chromophobe, and unclassified) are based on the number of patients with that subtype.

b The 95% CI was calculated using the two-sided Clopper-Pearson method.

c Durable stable disease = duration ≥23 wk.

d Clinical benefit rate = complete response + partial response + durable stable disease.

e Disease control rate = complete response + partial response + stable disease.

f Median PFS and OS were estimated using the Kaplan-Meier product-limit method and the 95% CIs were estimated with a generalized Brookmeyer and Crowley method.

g Given the small sample size, results should be interpreted with caution.

**Table 3 –:** Treatment-emergent adverse events (TEAEs) occurring in ≥15% of the 31 patients

TEAE, Preferred Term ^a^	Patients, *n* (%)	
	Any grade	Grade ≥3

Fatigue	22 (71)	2 (6)
Diarrhea	18 (58)	3 (10)
Decreased appetite	17 (55)	0
Nausea	17 (55)	2 (6)
Vomiting	16 (52)	2 (6)
Stomatitis	12 (39)	0
Weight decreased	12 (39)	0
Hypertension	10 (32)	5 (16)
Abdominal pain	9 (29)	1 (3)
Dyspnea	8 (26)	0
Epistaxis	8 (26)	0
Headache	8 (26)	0
Insomnia	8 (26)	0
Proteinuria	8 (26)	2 (6)
Arthralgia	7 (23)	0
Back pain	7 (23)	1 (3)
Dysphonia	7 (23)	0
Anxiety	6 (19)	0
Blood creatinine level increased	6 (19)	0
Constipation	6 (19)	0
Dyspepsia	6 (19)	0
Nasal congestion	6 (19)	0
Cough	5 (16)	0
Dizziness	5 (16)	0
Hypothyroidism	5 (16)	0
Malignant neoplasm progression	5 (16)	4 (13)
Muscular weakness	5 (16)	0
Pruritus	5 (16)	0

a Adverse event terms were coded using Medical Dictionary for Regulatory Activities version 22.0 and graded using Common Terminology Criteria for Adverse Events version 4.03.

## Appendix A. Supplementary data

Supplementary material related to this article can be found, in the online version, at doi:https://doi.org/10.1016/j.eururo.2021.03.015.
