# Supplementary material for: A Single-arm, Multicenter, Phase 2 Study of Lenvatinib Plus Everolimus in Patients with Advanced Non-Clear Cell Renal Cell Carcinoma
Source: Eur Urol. Author manuscript; Available in PMC 2025 Dec 8. (PMC12684810; doi:10.1016/j.eururo.2021.03.015)
Supplement: Supplementary Material [file NIHMS2094701-supplement-Supplementary_Material.pdf]

Supplementary Figure 1. Percentage Change in Total Sum of Target Lesion Diameters From Baseline to Postbaseline Nadir per RECIST v1.1, by Independent Imaging Review[a]

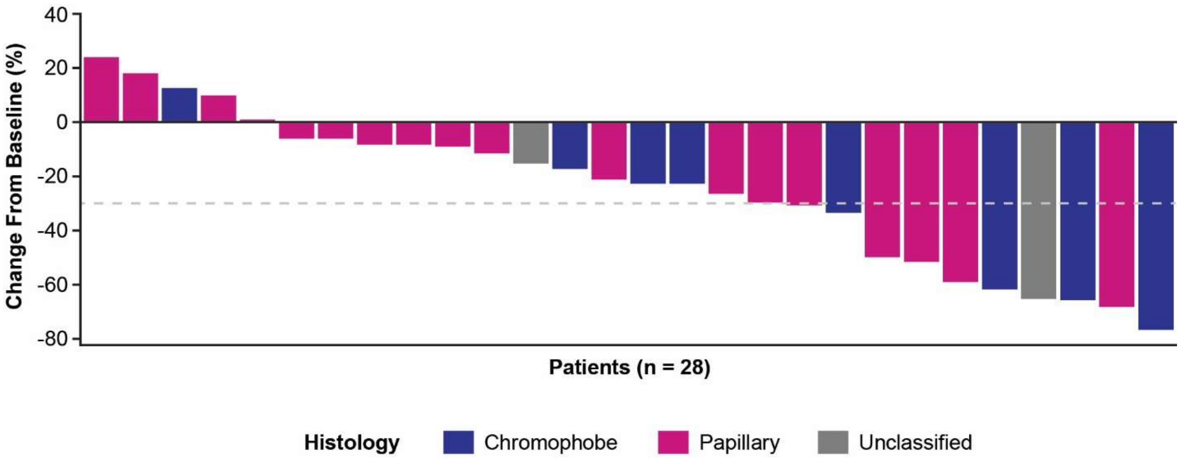

[a]This analysis included patients with both baseline and at least one postbaseline target lesion assessment.
RECIST v1.1, Response Evaluation Criteria In Solid Tumors version 1.1.
